# A systematic review of methods to assess intake of saturated fat (SF) among healthy European adults and children: a DEDIPAC (Determinants of Diet and Physical Activity) study

**DOI:** 10.1186/s40795-018-0231-1

**Published:** 2018-05-08

**Authors:** Fiona Riordan, Roisin McGann, Ciara Kingston, Ivan J. Perry, Matthias B. Schulze, Lene Frost Andersen, Anouk Geelen, Pieter van’t Veer, Simone J. P. M. Eussen, Martien C. J. M. Van Dongen, Nicole E. G. Wijckmans-Duysens, Janas M. Harrington

**Affiliations:** 1 School of Public Health, University College Cork, Western Road, Cork, Ireland; 2 School of Public Health, Physiotherapy and Population Science, University College Dublin, Dublin, Ireland; 3 School of Food and Nutritional Sciences, University College Cork, Cork, Ireland; 4 Department of Molecular Epidemiology, German Institute of Human Nutrition Potsdam-Rehbruecke, Nuthetal, Germany; 5 Department of Nutrition, Institute of Basic Medical Sciences, University of Oslo, Oslo, Norway; 6 Division of Human Nutrition, Wageningen University and Research, Wageningen, Netherlands; 7 Department of Epidemiology of the Faculty of Health, Medicine and Life Sciences, Maastricht University, Maastricht, The Netherlands

**Keywords:** Saturated fat, Dietary assessment, Europe, DEDIPAC

## Abstract

**Background:**

Dietary fat is an essential macronutrient. However, saturated fact has been associated with negative health outcomes including cardiovascular disease. Shifting consumption from saturated fat to unsaturated fats and limiting the level of saturated fat in the diet has been recommended. Currently, there is no standard method to measure saturated fat intake in etiologic studies. Therefore, it is difficult to obtain a reliable picture of saturated fat intake in Europe. To inform the development of the DEDIPAC (DEterminants of DIet and Physical Activity) toolbox of methods, we aimed to identify the assessment methods and specific instruments which have been used to assess saturated fat intake among children or adults in pan-European studies.

**Methods:**

Three electronic databases were searched for English language studies of any design which assessed intake of saturated fat. Reference lists were hand-searched. Studies were included if they were conducted in two or more European countries, and involved healthy, free-living children and adults.

**Results:**

The review identified 20 pan-European studies which assessed saturated fat intake. Food Frequency Questionnaires (*n* = 8) and diet records (*n* = 7) were most common, followed by 24-h recalls (*n* = 5). Methods differed in portion size estimation and the composition data which was used to calculate nutrient intake. Of the instruments used in more than two European countries, five Food Frequency Questionnaires had been specifically tested for validity to assess saturated fat intake; four among adults (Food4me, PURE, IMMIDIET, Health, Alcohol and Psychosocial factors in Eastern Europe (HAPIEE)) and one among children (used by Piqueras et al.).

**Conclusions:**

A standardised approach to portion size estimation and a common source of food composition data are required to measure saturated fat intake across Europe effectively. Only five instruments had been used in more than two European countries and specifically tested for validity to assess saturated fat intake. These instruments may be most appropriate to evaluate intake of saturated fat in future pan-European studies. However, only two instruments had been tested for validity in more than one European country. Future work is needed to assess the validity of the identified instruments across European countries.

**Electronic supplementary material:**

The online version of this article (10.1186/s40795-018-0231-1) contains supplementary material, which is available to authorized users.

## Background

Dietary fat is an essential macronutrient, providing a source of energy and facilitating the absorption of fat-soluble dietary components such as vitamins [1]. Saturated fatty acids (SFA) have been associated with the development of non-communicable diseases, including cardiovascular disease (CVD) [2–4]. The World Health Organisation (WHO) Global Strategy on Diet and Physical Activity recommends shifting consumption from saturated fat (SF) to unsaturated fats, and limiting the level of SF in the diet [5]. The Food and Agriculture Organisation expert consultation on fat and fatty acids in human nutrition has proposed that SFA be replaced by Monounsaturated Fatty Acids (MUFA) and Polyunsaturated Fatty Acids (PUFA) in the diet to reduce the risk of Coronary Heart Disease [4].

The role of SF in the diet has recently been the subject of debate. Some studies suggest SF increases levels of beneficial high-density lipoprotein (HDL). However, whether this offsets the effect of detrimental low-density lipoprotein (LDL), and consequently the risk of CVD, is unclear [6, 7]. To better understand the role of SF in the development of chronic disease there is a need for dietary assessment methods which can measure SF and its contribution to daily energy intake in a reliable and consistent way. However, a number of factors have made cross-country comparisons of macronutrient intake difficult: differences in the methods used to assess dietary intake, different approaches to portion size estimation, and the type of food composition databases (FCD) used to calculate SF intake.

In recent years there has been growing emphasis on the standardisation of food classification systems, including Food Composition Databases (FCD), between European countries. This has been the focus of a number of European projects [8–15], including The Innovative Dietary Assessment Methods in Epidemiological Studies and Public Health (IDAMES) project, which aims to develop new methods to assess dietary intake in Europe [16]. The European Food Safety Authority (EFSA) has recommended the standardized 24-HDR recall method, EPIC-Soft (now known as GloboDiet) [17, 18]. However, there are no agreed standards with respect to the assessment of macronutrients, including SF, for monitoring purposes or aetiological studies.

Partly in recognition of the lack of agreed standards and methodologies, the DEDIPAC: “DEterminants of DIet and Physical Activity” project [19], aimed to create a toolbox of dietary assessment methods which may be most appropriate to use in pan-European studies [19, 20]. The purpose of the current systematic literature review is to identify the assessment methods and specific instruments which have been used to measure intake of SF in European children or adults in more than one European country.

## Methods

### Data sources and study selection

This review adheres to the guidelines of the Preferred Reporting Items for Systematic Reviews and Meta-Analyses (PRISMA) Statement. The protocol for the review can be accessed from the PROSPERO (CRD42014014175) [21]. A systematic literature search was conducted for pan-European studies which assessed the intake of SF. SF are fatty acids where the fatty acid chain have predominantly single bonds. They can be classified as short, medium, long and very long chain, and are mainly provided in the diet by animal dairy fats, along with some oils, palm oil and coconut oil [4]. Three databases, PubMed, EMBASE and Web of Science, were searched by FR and RM. Search terms included terms for fats (e.g. dietary fat/s, saturated fat/s, dietary fatty acid/s, saturated fatty acid/s, volatile fatty acid/s, non-essential fatty acid/s, trans fatty acid/s, short chain fatty acid/s, trans fat/s, animal fat/s, lipid/s), along with keywords for dietary and caloric intake, and terms for European countries. A full copy of the EMBASE search strategy is included in Additional file 1: Figure S1. All searches were limited to literatures in English published from 1990 through to 15th March 2017.

**Fig. 1:**
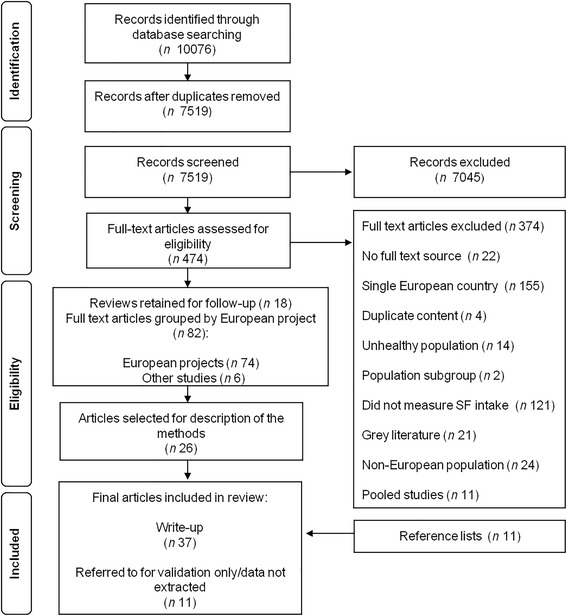
Flow diagram showing study selection process for the review

Titles and abstract screening of the articles was conducted by FR and RM. In the event of any uncertainty regarding inclusion, the full text of an article was sourced and reviewed. If FR and RM disagreed on article inclusion during full text review then they consulted a third author, JMH. To be included, studies had to be published in a peer-reviewed scientific journal, conducted in two or more European countries, as defined by the Council of Europe [22], and report on the intake of macronutrient SF. Therefore, studies were excluded if they only reported on fat as a food product (e.g. fat-based spreads, fats and oils). SF intake had to be measured at the individual level. Therefore studies which assessed SF intake at the household level or through analysis of biological samples were excluded. Studies had to be conducted among free-living, healthy populations. If study participants were hospital-based or belonged to a disease or societal sub-group they were excluded. The review was not limited by study design; studies with baseline intervention data, and case-control studies where intake was measured in population-based controls, were included (Fig. 1).

Reference lists of all included papers were hand-searched for additional publications. The names of European projects listed in the DEDIPAC Inventory of Relevant European Studies, were also used to search the databases. If necessary, study authors were contacted to request a copy of full paper, or the instrument or questionnaire.

### Data extraction and quality assessment

A data extraction form was created and piloted. This form recorded the following information from included studies: design, number and names of European countries involved, sample size (total and number for each country), age range of the included population, the method used and its description (including frequency categories for Food Frequency Questionnaires (FFQs), details of nutrient intake assessment, details of portion estimation), mode of administration, and details on the validation or reproducibility. Double extraction on each article was carried out by FR and RM. If necessary, further information on the methods was obtained from reference list of the originally included articles.

In line with a previous review of methods to assess fruit and vegetable (F&V) intake [23], the aim of the current review was to identify instruments. Therefore the quality of each included article was not appraised as part of the current review. Instead, information from the appropriate validation study was extracted by MvD, SE and NW. For an instrument to be considered suitable to assess intake in a pan-European study it had to meet two criteria: 1. Tested for validity; 2.Used in more than two countries as part of the same study. These two countries had to represent at least one country from at least three of the Southern, Northern, Eastern, Western European regions as defined by the United Nations [24]. Table 1 shows the results of this assessment.

**Table 1:** Summary of all studies identified to assess saturated fat: design, population studied, and dietary assessment instruments used and details of validation and/or reproducibility. Studies were selected to be included in this review based on the following two criteria: (1) the instrument was tested for validity and (2) the instrument was used in more than two countries simultaneously which represent a range of European regions

Authors/Study	Population	Countries	Instrument(s)	Macronutrient intake	Tested for validity	> 2 countries/country range
Bondia-Pons et al. [35]	Adults/Men Age range not reported (*n* = 160)	5 (Denmark, Finland Germany, Italy, Spain)	3-day record	SFA (% energy) was calculated by converting food consumption into corresponding nutrient intake using validated nutrition software from each country Other macronutrients (% energy): Protein, Carbohydrates, Fat, MUFA, PUFA		X
EPIC [14, 36–39]	Adults 35–70 years (*n* = 519,978)	10 (Denmark, England, France, Germany, Greece, Italy, the Netherlands, Norway, Spain, Sweden)	FFQ^a^ 24-HDR	SFA (g/day) was calculated from 24-HDR data using EPIC Nutrient DataBase (ENDB) created to aid cross-country harmonisation of data [73] Other macronutrients: Total Fat, MUFA, PUFA Origin of fat: animal, plant, unknown/mixed F&V intake [23] SSBs intake [74]	X [63] Belgium, the Czech Republic, France, the Netherlands and Norway	X
Esteve et al. [40]	Adults/Controls Age range not reported (*n* = 2736)	4 (France, Italy, Spain, Switzerland)	FFQ	SFA (g/day) was calculated using an ad hoc food-composition table compiled by INSERM on the basis of those published by McCance and Widdowson and revised by Paul and Southgate Other macronutrients: Protein (Total / Animal / Vegetable), Carbohydrates, Fibers, Total lipids, MUFA, PUFA		X
Food Habits in Later Life [27, 41]	Adults/Elderly 70+ years (*n* = 453)	2 (Greece, Sweden)	FFQ	SFA (g/day) was calculated using validated nutrition software (NUTTAB 1995), which is based on Australian FCTs modified to include additional Greek dishes and Swedish foods Other macronutrients: MUFA, PUFA, Carbohydrate, Protein Food sources: Olives, Olive oil, Other oil, Butter, Margarine [As Swedish data did not have a separate oil/fats food group because they were included in various recipes/dishes, they were unable to identify the quantity of oil/fat used in dishes in order to generate this food group and thus excluded the Swedish data when analysing fat intake]		
Food4Me [42–44]	Adults (*n* = 5562) 17–79 years	7 (Ireland, the Netherlands Spain, Greece, UK, Poland, Germany)	FFQ (Web-based)	SFA (% total energy), SFA (g) Nutritional composition and portion sizes were calculated from the 2008–2010 National Adult Nutrition Survey (NANS) database [43] Other macronutrients: MUFA, PUFA, Carbohydrates, Protein F&V intake [23] SSBs intake [74]	X [42, 43] UK Comparison with EPIC-Norfolk FFQ using a sample of 117 participants [43] Reproducibility tested using test-retest (*n* = 100) on 2 occasions 4 weeks apart [42] Validated using a 4-day weighed food record [42]	X
HAPIEE [45]	Adults (*n* = 28,947)	3 (Russia, Poland, Czech Republic)	FFQ	Saturated fat (g/day). McCance and Widdowson’s FCD Other macronutrients (g/day): Total carbohydrate, Protein, Total fat, PUFA, Trans fat Food sources: Animal fats and oils	X [75] UK Based on the Whitehall II questionnaire. Validated against a 7-day diet diary and biomarkers of nutrient intake by Brunner et al. [75] Assessed by energy-adjusted correlations, mean or median differences, and exact level of agreement.	
IMMIDIET [31, 46]	Adults 26–65 years (*n* = 802)	3 (Belgium, England, Italy)	FFQ	Total saturated lipids (% kcal/day) was calculated using Nutrition Analysis of FFQ (NAF), a computer programme used to convert the questionnaire dietary data into frequencies of consumption and average daily quantities of foods (energy and nutrients consumed). NAF was linked to the McCance FCT, the Italian FCT for Epidemiological studies, and the Dutch NEVO and Flemish-Belgian Nubel FCTs SF sub-types: myristic (C14), plamitic (C16), stearic (C18), arachidic (C20) Other macronutrients: unsaturated fats Food sources (food product groups used in dietary pattern analysis) (g/day): Starches, Cabbages and root vegetables, Leafy vegetables and tomatoes, Fruit and fruit or vegetable juices, Dairies, Cheese, Pasta and rice, Red meat and processed products, White meat and products, Fishes, Mollusks, Vegetable oils, Olive oil, Nuts and pizza, Sweets and sugars, Snacks, Chocolate coffee and tea, Mayonnaise, Alcoholic drinks	X [31] Belgium. UK and Netherlands FFQ validated as part of the EPIC study. Belgian FFQ was validated by Van Dongen et al. [31]	X
North/South Food consumption project [34, 47]	Adults 18–64 years (*n* = 1379)	2 (Northern Ireland, Republic of Ireland)	7-day record	SFA (g/day), SFA (% energy), and % contribution of the major food groups to SFA, were calculated by analysing food using WISP, which used the McCance and Widdowson’s Composition of Foods 5th Edition, and also included additional data (including analysis of recipes of composite dishes, manufacturer’s data on Irish foods and new products). Other macronutrients (g): Protein, Fat, Carbohydrate, Total sugars Food sources (contribution to daily fat intake): Meat and meat products. Butter spreading fats and oils, Biscuit cakes pastries and puddings, Milk and yoghurt, Potatoes and potato products, Sugars preserves confectionary and savoury snacks, Vegetables and vegetable dishes including pulses, Breads and rolls, Cheese, Eggs and egg dishes, Fish and fish dishes, Flours grains starches rice pasta and savouries, Soups sauces and miscellaneous foods, Creams ice-creams and chilled desserts		
Parfitt et al. [48]	Adults/Students 18–32 years (*n* = 48)	2 (England, Italy)	5 or 7-day record	SF (% of total fat) was calculated by converting food consumption into corresponding nutrient intake with validated nutrition software from each country: England: Salford Microdiet version 7, a computer software based on and updated from McCance and Widdowson’s FCTs,Italy: Foodometer, computer software, using data from the Italian National Institute of Nutrition Other macronutrients: MUFA, PUFA		
PURE [32, 49]	Adults 30–70 years > 154,000 men and women in the PURE study on a whole.	3 European. 4 LIC: Zimbabwe, Bangladesh, India, and Pakistan; 10 MIC: South Africa, Brazil, Argentina, Colombia, Chile, Poland, China, Malaysia, Iran, and Turkey; and 3 HIC: Canada, Sweden, and UAE.	FFQ	SFA (g/day) was calculated from the Polish version of the FFQ using a master international nutrient database which has been created primarily based on the United States Department of Agriculture (Washington, D.C.) FCD and modified appropriately with reference to local FCT, and supplemented with recipes of locally eaten mixed dishes Other macronutrients: Protein, Fat, Carbohydrate, MUFA, PUFA	X [49] Poland. To validate the shortened FFQ, a convenience sample (*n* = 146) of the PURE study participants in an urban (*n* = 73) and rural (n = 73) setting were recruited. The FFQ administered twice, approx. 1 year apart.	X
Seven Countries Study [50, 51]	Adults/Men 40–59 years at enrolment 70–89 years at 30 year follow up [50] (n ≈ 11,500)	7 (Finland, Greece, Italy, Japan, The Netherlands, USA, Yugoslavia)	Cross-check dietary history method	SF (g) were calculated for each cohort using information obtained from buying food products representing the average daily intake in a cohort and analysing for composition of SF [51] SF (% energy) was calculated using computerised versions of local food tables [50] Other macronutrients: Protein (% energy), MUFA (% energy), PUFA (% energy), Carbohydrate (% energy), Fibre (g/1000 kcal) F&V intake [23]	Tested for reproducibility only. [64] The reproducibility of food intake was investigated in repeated surveys carried out three and 12 months after the initial survey, by Bloemberg et al. [64]	X
SENECA [52, 53]	Adults/Elderly (n ≈ 2600) 70–75 years	12 (Belgium, Denmark, France, Greece, Hungary, Italy, Netherlands, Norway, Poland, Portugal, Spain, Switzerland)	Modified dietary history method comprising a 3-day estimated record and meal-based frequency checklist	SFA (% energy) Foods were translated into nutrients by using country-specific food-composition tables [52] Other macronutrients: PUFA, Protein, Fibre F&V intake [23] SSBs intake [74]	X [65] Validated against a 3-day weighed record [65].	X
Van Diepen et al. [54]	Adults/Students Age range not reported (*n* = 185)	2 (Greece, the Netherlands)	24-HDR	SFA (% energy) was calculated using ‘Food Processor 7.40, a computer software of which had the addition of Greek and Dutch recipes. It used estimated levels of intake Other macronutrients: MUFA, PUFA, Fibre, Protein, Carbohydrates		
Van Oostrom et al. [55]	Adults 20–55 years (*n* = 94)	2 (The Netherlands, Spain)	3-day record	SF (g) and SF (% energy) were calculated using validated nutrition software from each country: The Netherlands: Dutch Nutrient Database Spain: Nutrition tables for Spain. Other macronutrients: Total fat, MUFA, PUFA, Carbohydrates, Protein		
WHO-MONICA EC/MONICA Project optional nutrition study [33, 56]	Adults 45–64 years (*n* = 7226)	9 (Northern Ireland (Belfast), UK (Cardiff), Denmark, Finland, Belgium, Germany, France, Italy, Spain) 4 (Sub-study comprising of Germany, Finland, France and Northern Ireland) 2 (Sub study comprising of France and Northern Ireland)	3-day and 7-day records	Food consumption was converted into corresponding nutrient intake using validated nutrition software from each country [76];SFA (g) were calculated by Evans et al... [56];SFA (% energy) was calculated by Winkler et al. [76] using local FCT from the 4 countries Other macronutrients: PUFA, MUFA, Carbohydrates, Protein		X
ZENITH [57]	Adults/Elderly 55–87 years (*n* = 387)	3 (France, Italy, Northern Ireland)	4-day recall method	SFA (g/day) was calculated using food consumption data were converted into energy, macro- and micronutrients using relevant FCTs for each country. For foods and nutrients not available in the FCTs, these were assigned the compositions of similar foods derived from other databanks Other macronutrients: Total fat, MUFA, PUFA, Carbohydrates, Fibre, Protein		X
HELENA [28, 29, 58, 59]	Adolescents 12–17 years (*n* = 3000)	9 (Austria, Belgium, France, Germany, Greece, Hungary, Italy, Sweden, Spain)	24-HDR	SFA (% of total fat) was calculated by using the German Food Code and Nutrient Data Base (Bundeslebensmittelschlussel, BLS, Version II.3.1) [59] Other macronutrients: Carbohydrates, Protein, Fat, PUFA, MUFA, Fibre Correlations between food groups and fatty acid intake (SFA, PUFA and MUFA) examined [59] F&V intake [23] SSBs intake [74]	X [58, 67] 24-HDR evaluated by Vereecken et al. [58, 67] Intakes of energy and eight nutrient components calculated using different FCTs approaches were compared [66]	X
EYHS [60]	Children 9 years, 15 years (*n* = 2182)	2 (Sweden, Estonia)	24 HDR supplemented with a 2-day record	SF (% energy) was calculated using validated nutrition software from each country: Sweden: Nutrient intake data were analysed in Sweden using the Swedish FCD PC-kost (maintained by the Swedish National Food Administration), Estonia: The Finnish FCD Micro-Nutrica 2.0 (modified and translated into the Estonian language at Tallinn University of Technology, Department of Food Processing) Other macronutrients: Protein, Carbohydrate, Fat, MUFA, PUFA, Fibre		
IDEFICS [30, 61]	Children (n = 16,864) 2–9 years [14,972 children proxies completed the FFQ. 14,863 recall interviews completed]	8 (Belgium, Cyprus, Estonia, Germany Hungary, Italy, Spain, Sweden)	FFQ 24-HDR	Linking to country-specific FTCs, and using the SACINA data daily intake (g/day) of all food groups the ratio of unsaturated to saturated fats was calculated [61] F&V intake [23] SSBs intake [74]	X [67–71] SACINA is based on the YANA-C instrument validated as part of the HELENA study by Vereecken et al. [67] SACINA was validated by Börnhorst et al. [68] using the doubly labelled water technique The CEHQ-FFQ has been validated using 2 24-HDRs [71] The reproducibility of CEHQ-FFQ has been examined by Lanfer et al. [69], and the validity by Huybrechts et al. [70]	X
Piqueras et al. [62]	Children (*n* = 176) 4 years	3 (Spain, Germany, Hungary)	FFQ	SF (g) and SF (% energy) were calculated using the program CESNID and the database used for the nutrient analysis was the 3rd edition of CESNIDs food tables	X [72] Sweden FFQ based on one used in MONICA which was validated by Johanasson et al. [72]	X

*FFQ* Food Frequency Questionnaire, *24-HDR* 24 h dietary recall, *F&V* Fruit and Vegetable, *SSBs* Sugar-Sweetened Beverages, *MUFA* Monounsaturated Fatty Acids, *PUFA* Polyunsaturated Fatty Acids, *EPIC* European Prospective Investigation into Cancer and Nutrition, *EYHS* European Youth Heart Study, *HELENA* Healthy Lifestyle in Europe by Nutrition in Adolescence, *HAPIEE* Health, Alcohol and Psychosocial factors in Eastern Europe, *IDEFICS* Identification and prevention of Dietary- and lifestyle-induced health EFfects In Children and infantS, *PURE* Prospective Urban Rural Epidemiology, *SENECA* Survey in Europe on Nutrition and the Elderly; a Concerted Action, *WHO-MONICA* The World Health Organization Multinational MONItoring of trends and determinants in CArdiovascular disease, *ZENITH* Zinc Effects on Nutrient/nutrient Interactions and Trends in Health and Aging

^a^EPIC FFQs were country-specific, and as a result are not discussed in the review

## Results

### Description of the included studies

In total 10,076 papers were identified. After removing duplicates 7519 remained. Following title and abstract screening and full text review, 82 primary research articles were retained. These articles were organised by the European project to which they belonged. If they did not belong to a project they were grouped as ‘Other’ (*n* = 6) (see Fig. 1 for a breakdown). ‘Study’ refers to the larger project, rather than individual articles based on the same project and methodology. Of the 82 articles retained, 26 provided a detailed description of the project or the method in question. These 26 articles were selected for the current review, which equated to 1–3 articles per project. A further 11 articles were sourced from reference lists [14, 25–34].

In total, 37 articles [14, 27–62] from 20 studies were included in the review. This number included articles from the original search (*n* = 26), and from reference lists (*n* = 11). Articles in which the instrument was tested for validity were also recorded (*n* = 11) [58, 63–72]. The characteristics of the included studies are described in Table 1. They comprised of large pan-European studies (*n* = 11) and smaller studies conducted in 2–4 countries (*n* = 6). Four studies assessed intake of SF in children [60–62], or adolescents [59], and 13 assessed intake among adults [35, 40, 41, 46–50, 54–57, 73].

### Dietary assessment methods

#### Types of methods

Four approaches to measure SF intake were identified: FFQs, 24 h recalls (24-HDRs), dietary record/diet diaries, or dietary history methods. Most studies used FFQs or 24-HDRs. Several studies used instruments which had been tested for validity: IMMIDIET, European Prospective Investigation into Cancer and Nutrition (EPIC), Prospective Urban Rural Epidemiology (PURE), Healthy Lifestyle in Europe by Nutrition in Adolescence (HELENA), and Identification and prevention of Dietary- and lifestyle-induced health EFfects In Children and infantS (IDEFICS). One study instrument was based on a FFQ which had been used as part of a different study and previously tested for validity [62]. Countries in the EPIC study did not use a common FFQ instrument, therefore only the EPIC-Soft instrument is discussed in this review.

According to the two criteria (Table 1) six study instruments were appropriate to assess intake of SF in future pan-European studies. Two, the EPIC 24-HDR instrument EPIC-Soft, and the cross-check dietary history method used by the Seven Countries Study, had been used to measure intake among adult populations. The HELENA-DIAT instrument had been used among adolescents. The IDEFICS FFQ and 24-HDR, and the FFQ used by Piqueras et al. [62] had been used among children. Table 1 shows the instruments which met the two criteria. Some of these instruments were also used to assess intake of other macronutrients, and/or also met the criteria to assess intake of F&V [23] or Sugar Sweetened Beverages (SSBs) [74] as determined from two previous reviews. This is also indicated in Table 1.

### Validation

Of the studies which assessed instrument validity and thus fulfilled inclusion criterion 1 (Table 1), only two instruments, EPIC-Soft [63] and the IDEFICS 24-HDR [68], had been tested for validity in more than one country: Belgium, the Czech Republic, France, the Netherlands and Norway in the case of EPIC-Soft [63]; Belgium and Spain in the case of the IDEFICS 24-HDR [68].

Five instruments, all FFQs [31, 42, 43, 49, 72, 75], had been tested specifically for validity to assess SF intake, in Sweden (Piqueras et al. [62]), UK (Food4Me [42, 43]; Health, Alcohol and Psychosocial factors in Eastern Europe (HAPIEE) [45]), and Poland (PURE [49]), and Belgium (IMMIDIET [31]). FFQs [43], repeated 24-HDRs [31, 49, 72] or diet/food records [42, 75] were used as reference methods. Validity was assessed by crude correlations [31, 42, 49, 72], de-attenuated correlation coefficients [49, 72], mean or median differences in SF consumption [49, 72, 75], exact level of agreement of SF consumption [31, 49, 75], or Bland and Altman’s plots [31, 42, 43, 49].

In four studies, the instrument reproducibility was also tested. Reproducibility was assessed by correlations [43, 49, 72], mean/median differences, or intraclass correlation coefficient (ICC) [49] between subsequent assessments of FFQs. References for validation studies are provided in Table 1. Where available, results of the statistical assessments are provided in Table 2.

**Table 2:** Information on the validity and reliability of SF consumption

	Validity			Reliability			
Instrument/Author	Correlation	Mean/median differences	Exact level of agreement, %	Correlation	Mean/median differences	Exact level of agreement, %	Interval between 2 subsequent assessments Comments
Food4Me FFQ Fallaize et al. 2014 [42]	Food4Me FFQ vs. 4-day WFR Pearson’s crude 0.48	Food4Me FFQ 45.6 g (SD 15.6) 4-day WFR 24.3 g (SD 10.4)	Exact agreement: 37	Pearson ‘s crude 0.81	Food4Me FFQ1: 33.2 g (SD 14.2) Food4Me FFQ2: 32.2 g (SD 14.5)	Exact agreement: 60	4 weeks apart
Food4Me FFQ Forster et al. 2014 [43]	Food4Me FFQ vs. EPIC Norfolk FFQ Pearson’s crude 0.71	Food4Me FFQ 36.0 g (SD 16.5) EPIC Norfolk FFQ 24.9 g (SD 10.5)	Exact agreement: 46	NA	NA	NA	NA
HAPIEE FFQ Brunner et al. 2001 [75]	HAPIEE FFQ vs. 7-DD Spearman’s rank Men: 0.43 Women: 0.56	7DD Men: 38 g (SD 13) Energy adjusted 38 g (SD 9) Women: All: 30 g (SD 11) Energy adjusted 30 g (SD 7) HAPIEE FFQ Men: All 33 g (SD 14) Energy adjusted 33 g (SD 8) Women: All 28 g (SD 12) Energy adjusted 28 g (SD 8)	Exact agreement between 7DD, FFQ and biomarker concentrations Men 7DD: 33 FFQ: 28 Women 7DD: 37 FFQ: 38 Exact agreement between 7DD and FFQ: Men: 40 Women: 41	NA	NA	NA	NA
IMMIDIET FFQ Van Dongen et al. 2011 [31] *Validation: FFQ* vs. *five 24 h-dietary recalls*	IMMIDIET-FFQ vs. 24-h DR Pearson’s, crude men: 0.21 women: 0.34	IMMIDIET-FFQ *men:56 g. (SD 22 g.)* *Women:* *46 g. (SD 19 g.)* 24-h DR*men: 33 g. (SD 9 g.)* *women: 25 g. (SD 8 g.)*	Exact agreement: men: 37 women: 26	NA	NA	NA	NA
Northern Sweden FFQ (used by Piqueras et al) Johansson et al. 2002 [72] *Validation: 2 FFQ’s and ten 24-h recalls* *Reproducibility:2 FFQ’s over a one-year interval*	Northern Sweden FFQ vs. 24-h DR Pearson’s, crude men: 0.58 women: 0.54 Pearson’s, de-attenuated men: 0.62 women: 0.59	Northern Sweden FFQ men: 31.6 g. women: 23.4 g. 24-h DR men: 34.8 g. women: 26.6 g.	NA	Northern Sweden FFQ1 vs. FFQ2 Pearson’s, crude men: 0.59 women: 0.70	NA	NA	one year
PURE FFQ Dehghan et al. 2012 [49] *Validation: 2 FFQ’s and four 24-h recalls* *Reproducibility: 2 FFQ’s over a one-year interval*	FFQ1 vs. DR Pearson’s, crude Urban area: 0.46 Rural area: 0.39 FFQ2 vs. DR Pearson’s, crude Urban area: 0.42 Rural area: 0.33 FFQ1 vs. DR Pearson’s, de-attenuated Urban area: 0.63 Rural area: 0.60 FFQ2 vs. DR Pearson’s, de-attenuated Urban area: 0.58 Rural area: 0.50	Dietary Recalls Urban area: 25.1 g. Rural area: 20.7 g. FFQ1 Urban area: 25.0 g. Rural area: 36.7 g. FFQ2 Urban area: 22.6 g Rural area: 35.2 g	Exact agreement FFQ1 Urban area: 87,7 Rural area: 74.0 FFQ2 Urban area: 80.8 Rural area: 63.0	FFQ1 vs. FFQ2 Pearson’s, crude Urban area: 0.54 Rural area: 0.40 ICC Urban area: 0.54 Rural area: 0.38	NA	NA	one year

*FFQ* Food Frequency Questionnaire, *24-HDR* 24 h dietary recall, *WFR* weighed food record, *DD* Diet Diary, *DR* Diet Record, *ICC* intraclass correlation coefficient, *EPIC* European Prospective Investigation into Cancer and Nutrition, *HAPIEE* Health, Alcohol and Psychosocial factors in Eastern Europe, *PURE* Prospective Urban Rural Epidemiology

### Instruments tested for validity

Details of the five instruments, HAPIEE, Food4Me, PURE, IMMIDIET FFQs, and the FFQ used by Piqueras et al.*,* are summarised in Table 3. The HAPIEE FFQ had moderate agreement (0.4–0.6) for SF intake with a 7-day diet diary (7DD) (Spearman’s rank correlation: men, *r* = 0.43; women, *r* = 0.56) [75], as did the Food4Me FFQ which was tested for validity using a 4-day weighed food record (Pearson’s crude correlation: *r* = 0.48) [42]. The Food4Me FFQ also had good agreement (> 0.6) with the EPIC Norfolk FFQ (*r* = 0.71) [43]. The FFQ used as part of the PURE study had low to moderate agreement with 4 repeated 24-HDR (urban: *r* = 0.42; rural: *r* = 0.39) [49] whereas the IMMIDIET FFQ had low agreement (< 0.4) with five repeated 24-HDR (men: *r* = 0.21; women = 0.34) [31]. Of the instruments used among children, the FFQ used by Piqueras et al. had good to moderate agreement with 10 repeated 24-HDRs (men: *r* = 0.58; women: *r* = 0.54) [72].

**Table 3:** Summary of the instruments which were validated (*n* = 5) for assessment of saturated fat

Study/Instrument	Design	Age group	Countries	Mode	Portion estimation
Adults					
HAPIEE [45] FFQ Validation: 7-day diet diary [75]	Cross-sectional	45–69 years	3 (Russia, Poland, Czech Republic)	Self-admin	X
IMMIDIET FFQ [31, 46] Validation: 5 24-HDRs [31]	Cross-sectional	26–65 years	3 (Belgium, England, Italy) also in Table 1	Self-admin.	X
PURE FFQ [32, 49] Validation: 2 FFQ’s and 4 24-HDRs [49]	Cross-sectional	30–70 years	3 European 4 LIC: Zimbabwe, Bangladesh, India, Pakistan; 10 MIC: South Africa, Brazil, Argentina, Colombia, Chile, Poland, China, Malaysia, Iran, Turkey; and 3 HIC: Canada, Sweden, UAE	Self-admin	X
Food4Me FFQ [42–44] Validation: 4-day weighed food record [42]	RCT	17–79 years	7 (Ireland, the Netherlands, Spain, Greece, UK, Poland, Germany)	Self-admin	X
Children					
Piqueras et al. FFQ [62] Validation: 2 FFQs and 10 24-HDR [72]	Cross-sectional	4 years	3 (Spain, Germany, Hungary)	Self-admin.	X

*FFQ* Food Frequency Questionnaire, *24-HDR* 24 h dietary recall, *HAPIEE* Health, Alcohol and Psychosocial factors in Eastern Europe, *PURE* Prospective Urban Rural Epidemiology

### Macronutrient assessment

The identified instruments measured the intake of SFA or SF in grams [42, 50, 55, 56, 62], grams/day [34, 40, 41, 45, 49, 57] or % contribution to daily energy intake [34, 35, 42, 46, 50, 53–55, 60, 62, 76], % of total fat [48, 58], and ratio of unsaturated to saturated fats [61]. Of the five instruments which were tested specifically for validity to assess SF, two reported SF as grams/day (HAPIEE [45], PURE [32, 49]), two as grams (Piqueras et al. [62], Food4Me [42–44]), and three as % energy/day (IMMIDIET [31, 46], Piqueras et al. [62], Food4Me [42–44]).

EPIC used the EPIC Nutrient DataBase (country-specific food composition data standardised across countries) to calculate intake of SF from dietary data [73]. Mulder et al. [51] (Seven Countries Study), determined SF intake by buying food products which represented the average daily intake in a cohort and analysing these products for composition of SF [51]. The remaining studies used local food composition tables (FCT) from participating countries to calculate intake. In some cases, one FCT was used as the main source of composition data. For example, the German Food Code and Nutrient Data Base (Bundeslebensmittelschlussel) was used by the HELENA study and supplemented with information from the Belgian FCT [59, 77].

### Food frequency questionnaires (FFQs)

Characteristics of the FFQ instruments are summarised in Table 4. Where this information was available, the number of FFQs items ranged from to 43 to 322. Most FFQs recorded habitual consumption over the previous year, with the exception of the IDEFICS FFQ [30, 61]. This FFQ assessed intake over a typical week during the previous month. Almost all FFQs were paper-based and self-administered. All were semi-quantitative, and assessed portion size either through specifying a standard portion size on the FFQ for the food item in question [41, 49], or asking participants to consult photos [46], or use household measures [78]. The number of pre-coded frequency categories on the FFQs ranged from 3 to 11.

**Table 4:** Summary of FFQs and their characteristics

Authors/Study	Type/# items	Purpose	Population	Ref. period	Mode	Categories	Portion estimated? (Yes/No)
Adults							
Esteve et al. [40]	Semi-quantitative dietary questionnaire	Test association between diet and cancers of the larynx and hypopharynx	Adults Age range not reported	12 months	Face-to-face interview	Structured by meals, i.e., breakfast, lunch, dinner, as well as early morning, mid-morning, mid-afternoon and late evening snacks	Yes. Assessed separately. Usual portion size estimated during interview
Food Habits in Later Life [27, 41]	Semi-quantitative FFQ # not reported	Test association of food habit with good health	Adults/Elderly 70+ years	12 months	Face-to-face interview	3 categories Ranging from daily to weekly and monthly	Yes Assessed in line Portion sizes were specified in units thought to be the most appropriate for the given food
Food4Me [42–44]	Semi-quantitative Web-based 157-item FFQ.	Determine impact of personalised dietary advice on eating patterns and health outcomes	Adults 18–79 years	Previous month	Self-administered	9 categories. Ranging from ‘Never or less than once a month’, to ‘5–6 times per day’, and ‘> 6 times per day’	Yes 3 photographs representing small, medium, and large portions. Participants could select one of the following options: very small, small, small/medium, medium, medium/large, large, or very large which were linked electronically to portion sizes (in grams)
HAPIEE [45]	Semi-quantitative Czech = 136-item FFQ Russian = 147-item FFQ Polish = 148-item FFQ	Test association between socio-economic indicators and diet	Adults 45–69 years	Previous three months	Interview (Russia & Poland) Self-administered. (Czech Republic)	9 categories. Ranging from ‘Never’ to ‘Six or more times per day’ Open-ended section where they could add any further foods not listed	Yes Assessed in-line A country-specific portion size for each food was specified Participants were asked how often, on average, they had consumed a ‘medium serving’ of the items – defined as about 100 g or 50 g depending on the food in question
IMMIDIET [31, 46]	Semi-quantitative 322- item EPIC-Italy FFQ (as above) EPIC-UK FFQ (as above)	Identify determinants (diet, genetic) of risk of myocardial infarction Determine role of dietary patterns in plasma and red blood cell fatty acids variation	Adults 26–65 years	12 months	Self-administered	9 categories. Ranging from ‘Never/rarely’; ‘1–3 days/month’ to ‘1,2,3,4,5,6,7 days per week’	Yes Assessed separately Recorded as absolute weights or as household measurements Photo book to estimate small, average, and large portions for spreads, bread spreads, and milk in coffee and tea
PURE [32, 49]	Semi-quantitative FFQ (country-specific) [Long Polish FFQ = 153-item [Short Polish FFQ = 134-item]	Examine the impact of societal influences on chronic non-communicable diseases	Adults 30–70 years	12 months	Self-administered	[based on copy of Polish questionnaire] 9 categories: Ranging from ‘Never’ to ‘> 6/day’	[based on copy of Polish questionnaire] Yes Assessed in-line Participants asked to report frequency of consumption of an average portion e.g. in case of ‘Butter’ (2 heaped Tbs), ‘Margarine’ (1 Tsp)
Children							
IDEFICS [30, 61]	Non-quantitative. 43-item FFQ	Determine the aetiology of overweight, obesity and related disorders	Children 2–9 years (parents or guardians as proxies)	Typical week over the previous month	Self-administered	8 categories. Ranging from ‘Never/less than once a week’ to ‘4 or more times per day’. ‘I have no idea’ was also an option.	No.
Piqueras et al. [62]	Semi-quantitative FFQ # items not reported	Examine association of dietary habits and child size	Children 4 years	Not reported	Self-administered	Not reported	Yes. FFQ described portion size for adults but they used portion sizes appropriate for children as informed by diet record data collected for similar aged children in the UK

*FFQ* Food Frequency Questionnaire, *HELENA* Healthy Lifestyle in Europe by Nutrition in Adolescence, *HAPIEE* Health, Alcohol and Psychosocial factors in Eastern Europe, *IDEFICS* Identification and prevention of Dietary- and lifestyle-induced health EFfects In Children and infantS, *PURE* Prospective Urban Rural Epidemiology

### Diet records

Six studies used diet records or diaries (Table 5) to assess SF intake. Portions were estimated using a photo book [33, 34, 50, 57], during the interview (portion description provided by a dietician) [55], using food models [33, 50], or by weighing foods [34, 48, 76]. Most of the identified records were three or seven day records, with one exception, the Zinc Effects on Nutrient/nutrient Interactions and Trends in Health and Aging (ZENITH) study. This study used a four recall day method, over two weekday and two weekend days. All records were self-administered.

**Table 5:** Summary of diet records and their characteristics

Authors/Study	Population	Purpose	Time period	Mode	Structure	Portion estimation
Bondia-Pons et al. [35]	Adults Age range not reported	Examine association of olive oil consumption with lipid profile and blood pressure	3 consecutive days	Self-administered	No details	No details
Parfitt et al. [48]	Adults/students 18–32 years	Examine dietary intake and anti-oxidant status	5 days or 7 days	Self-administered	No details	Yes Estimated and weighed. All portions eaten and component ingredients where relevant, were weighed on household scales For meals eaten out, portions were quantified in household measures, in some cases using the standard reference work Food Portion Sizes’17′ to help estimate portion sizes
North/South Food consumption project [34, 47]	Adults 18–64 years	Determine estimates of intake of dietary fibre and non-starch polysaccharide Establish a database of habitual food and drink consumption	7 days	Self-administered	Participant reported the types and amounts of all foods, beverages and nutritional supplements consumed over the 7-day period, and also the time and location of each eating occasion’, the method of cooking and brand name (where appropriate), leftovers, recipe details They also included their perceived definition of the ‘eating occasion’ either a meal or a snack Detailed instructions were given on the recording of recipes and food/drink eaten out	Yes Estimated and weighed. Respondents were asked to describe food quantities that they had eaten using an album of food photographs Fieldworkers obtained the weights of certain foods in the respondents’ homes using portable food scales For some foods, the amounts eaten were obtained from weights printed on food packaging. Manufacturers’ information was then added to an Extended Menu Search (EMS®) facility on the nutritional analysis program (WISP®, Tinuviel Software, Warrington, UK), which interfaced with the food diary data entry system (WISP-DES®, Tinuviel Software, Warrington, UK)
SENECA [52, 53]	Adults/Elderly 70–75 years	Examine cross-cultural differences in nutrition and life-style factors Examine cross-cultural variations and changes in intake over time	3 consecutive days	Self-administered (3 day record) followed by face-to-face interview	1. Estimated diet record, structured by 8 meal periods 2. Frequency checklist	Estimated and weighed. Portion sizes recorded in household measures and checked by weighing Beverages portion size estimated using glasses or cups
Van Oostrom et al. [55]	Adults 20–55 years	Examine relationship of dietary habits and lipid profile	3 non-consecutive days	Self-administered	No details	Estimated The participants estimated their intake in a quantitative manner through instructions given by an allocated dietician and aided by a standardised portion size table
WHO-MONICA EC/MONICA Project optional nutrition study [33, 56]	Adults/Men 45–64 years	Dietary determinants of cardiovascular disease	3 consecutive days (Belgium, France, NI, Finland, Italy) 7 consecutive days (Germany, Denmark, UK) 3 consecutive 24-HDRs (Spain)	Self-administered Interview admin. or telephone administered for Barcelona.	Generally week and weekend days representative for the whole week were included Data collection carried out in several seasons. Participants recorded the preparation method, type of food or brand name, and recipes	Estimated In the Winkler et al. [76] study all records used weighing and household measures to determine portion size, with the exception of Belfast 3-day record which used precise weighing According to the report on the assessment methodology [33], three main approaches were used: Picture book and food models (France (Spain, Italy, Germany) Household measures (Germany, France, Spain, Finland, UK, Italy, Denmark) Standard units (Germany, France, Finland, Spain, UK, Italy, Denmark)
ZENITH [57]	Adults/Elderly 55–87 years	Describe intake and status of vitamin A, vitamin E and folate in the middle-aged and old-aged population	4-day (recall method)	Self-administered	Included 2 weekdays and weekend days. Participants recorded all foods and drinks consumed, describing the foods and portion sizes in as much detail as possible	Estimated Portion sizes related on standard portion sizes using visual book reference standard of foods (SU.VI.MAX, 1994; DietoMetro, 1999)

*MONICA* multinational MONItoring of trends and determinants in CArdiovascular disease, *ZENITH* Zinc Effects on Nutrient/nutrient Interactions and Trends in Health and ageing, *SENECA* Survey in Europe on Nutrition and the Elderly; a Concerted Action

### Dietary history

A dietary history approach is an interview-based approach used to record usual intake, asking an individual to recall a typical intake patterns, typically over a longer period (e.g. 6 months) [79]. The Seven Countries Study used a cross-check dietary history method conducted by face-to-face interview. The dietary history recorded diet intake in the month preceding the interview. This method had been tested for reproducibility, albeit not specifically to assess SF intake [64]. Usual food consumption pattern was recorded (i.e. foods consumed at breakfast, lunch, dinner and between meals) on a daily basis during week and weekend days. A list of all foods was compiled from this record. Interviewers then recorded what was eaten on a daily, weekly, or monthly basis. A checklist with an extensive number of foods was also used to record the frequencies and amount of foods consumed. Portion size was estimated using different approaches: Finland: photos; The Netherlands: portable scale; Italy: artificial models of different foods in Italy), and also weighed [51].

### 24 hour dietary recalls (24-HDRs)

In total, six 24-HDRs were identified, three of which were computerised [61, 73, 80]. Their characteristics are summarised in Table 6. Portion size was estimated for all of identified 24-HDRs, using household measures [80, 81] or photographs [60, 61, 80, 81]. EPIC-Soft, estimated portion sizes using six quantification methods. Two 24-HDRs were tested for validity: HELENA-DIAT [80], which was compared with 1-day food records [58] and tested for reproducibility across administrative modes (self-administration and by interview [67]), and IDEFICS Self-Administered Children and Infants Nutrition Assessment (SACINA) which was tested for validity using the doubly labelled water technique [68].

**Table 6:** Summary of 24-HDR and their characteristics

	Population	Purpose	Method of admin	Structure	Prompts	Portion size
Adults						
EPIC [25, 73]	Adults 30–70 years	Provide comparable food consumption data between several European countries	Computerised, face-to-face interview	1. ‘Quick list’ Chronological entry of all foods and recipes consumed during day 2. Foods are entered per meal 3. Each ‘quick list’ item is described and quantified	Yes Program mediated Checklist of foods which are easily forgotten is displayed on screen	Estimated 6 quantification methods including photos (2–6 portion sizes), shapes, household measurements, standard units, standard portions, volume method. If the portion size estimation methods is unknown this can be entered by the interviewer
Van Diepen et al. [54]	Adults/Students Age range not reported	Examine relationship between Mediterranean diet and obesity	Not reported	Not reported	Not reported	Not reported
Adolescents						
HELENA [58, 67, 80]	Adolescents 13–17 years	Assess food and nutrient intake	Computerised, self-administered	Divided into 6 meal occasions. For each occasion the user selects all food and beverages consumed from a standardised food list. Foods and beverages which are not included in the list can be added	Yes Program mediated When appropriate, a text box appears on the screen probing for food items which are often eaten in combination with other items (for example, chips, ‘Don’t forget mayonnaise/ ketchup etc.!’) When extreme amounts are entered; a warning is given i.e. zero values are not accepted. At the end of the 24-h recall, the program checks entries for occurrence of fruit, vegetables and sweets. If one of these items has not been entered, the adolescent is asked whether it really was not consumed	Estimated Information on quantities was gathered by using household measurements or pictures of portion sizes
Children						
EYHS [60]	Children 9 years 15 years	Examine personal, environmental, and lifestyle influences on cardiovascular risk factors	Face-to-face interview	A qualitative 1-day record completed at home with help from parents if needed A face-to-face interactive 24-HDR interview was performed on the following day	Differences between the interview data and the record data were discussed with the participant	Estimated Portion size was estimated using pictures of portion sizes
IDEFICS SACINA [61]	Children 2–9 years (parents or guardians as proxies)	Determine the aetiology of overweight, obesity and related disorders	Computerised, face-to-face interview Hungary: self-admin. 24-HDR at home	6 meal occasions	Yes Program mediated	Estimated Photos

*SACINA* Self-Administered Children and Infants Nutrition Assessment, *EPIC* European Prospective Investigation into Cancer and Nutrition, *HELENA* Healthy Lifestyle in Europe by Nutrition in Adolescence, *EYHS* European Youth Heart Study, *IDEFICS* Identification and prevention of Dietary- and lifestyle-induced health EFfects In Children and infantS

## Discussion

All four main assessment methods; FFQs, 24-HDRs, diet records/diaries and diet history methods have been used in pan-European studies to measure intake of SF. Of the 20 studies identified, most assessed intake of SF among adults (*n* = 16), and few measured intake among adolescents or children (*n* = 4). While FFQs were most common (*n* = 8), they differed in terms of the approach used to determine portion size and calculate macronutrient intake. Only one identified study, EPIC, used a standardised database as a source of food composition data.

If intake of fat sub-types such as SF and their relationship with disease are to be studied in a standardised way across European countries, it is essential to identify valid instruments. Six study instruments met two criteria (1.the instrument was tested for validity, and; 2. used in more than two European countries) to assess SF among adults in pan-European studies: the EPIC-Soft 24-HDR, HAPIEE, Food4Me, IMMIDIET, and PURE FFQs, and the SENECA 3-day record. However, only four of these, all FFQs (HAPIEE, IMMIDIET, PURE and Food4Me) had been specifically tested for validity to assess SF intake. Two of these (HAPIEE and Food4Me FFQs) were found to have moderate agreement with diet records. The PURE FFQ had low to moderate agreement with repeated 24-HDRs, and the IMMIDIET FFQ had low agreement with repeated 24-HDRs. Only one identified instrument had been used among adolescents, HELENA-DIAT, but this had not been tested for validity to assess SF intake. Finally, the 24-HDR and FFQ used by the IDEFICS study, and the FFQ used by Piqueras et al. [62] had been used to measure SF intake among children. Of the two, only the FFQ used by Piqueras et al. [62] had been tested for validity to assess SF intake. This instrument had good to moderate agreement with repeated 24-HDRs.

All instruments which had been tested for validity to assess SF intake, had done so using food records (4 and 7 day) [42, 75] or 24-HDRs [31, 49, 72] as the reference method. However, using these methods as a reference assumes they are superior in terms of assessing true SF intake. No specific biomarkers for SF exist, therefore, the validity of a 24-HDR to assess true intake cannot be determined. This raises an important question: whether the identified instruments are valid to specifically assess SF intake. Another important consideration is the fact that the level of macronutrient intake may be affected by the source of FCD used for calculations [82]. Ideally pan-European studies would use a common data collection instrument tested for validity, a common approach to portion size estimation, and a standardised source of composition data to calculate intake of SF.

As with previous reviews the results will contribute to the DEDIPAC toolbox of dietary intake assessment methods. The two criteria used in this review, are only an initial approach to identifying suitable instruments. Other factors, including the existing evidence with respect to instrument validity together with instrument feasibility, should be taken into consideration when deciding the appropriateness of an instrument to assess intake of SF in a pan-European population. Only two instruments had been tested for validity in more than one European country. To determine which instruments may be most appropriate, will require further work to test validity across countries. Most identified instruments were also included in two previous reviews on methods to assess intake of F&V [23] and SSBs [74]. Exceptions were the ZENITH 4-day recall method, PURE FFQ, and the FFQs used by Van Oostrom et al., and Piqueras et al. Overall these two reviews identified a greater number, and variety, of instruments. While this review was limited to pan-European studies, this is not to suggest that other instruments used as part of non-European studies, could not be used to assess intake across Europe.

The review has a number of strengths and limitations. A comprehensive search strategy identified all pan-European studies measuring intake of SF among children or adults, and the instruments used by these studies. In addition to searching databases, reference lists were hand-searched and study authors were contacted to identify further instruments. A copy of the instrument was sourced in order to accurately describe each instrument. Although the search was comprehensive, it is possible that all relevant articles were not identified. Furthermore, the search was limited to English-language papers. Where a copy of the original instrument or article could not be sourced, the description may be limited, although the results can still be used as a reference. The quality of the identified instruments was not assessed as part of this review. It is important to emphasize that the current review only provides an initial selection of instruments that may be most appropriate to assess SF across European countries. A decision on appropriateness will depend upon instrument validity, which requires further research. Not all SF may be detrimental to health [83, 84]. In light of this, a final limitation of the review may be the focus on total SF intake. Assessing different SFs or subgroups and their relation to health, and reviewing instruments which examine and report on these differences may be an important next step. The majority of the identified instruments evaluated SF as one class. Only one FFQ, used in the IMMIDIET study [31, 46], assessed the intake of SF by sub-types. Lastly, it is important to consider the fact that the identified instruments rely on available food composition data for analysis; the assessment of SF may lag behind changes in food production and composition. FFQs may need to be updated in line with such changes e.g. adding new foods, changing numbers on answer options.

## Conclusion

This review has identified a range of methods to assess intake of SF, FFQs being the most common method used. Key differences exist between the instruments which are currently available to assess SF intake. In order to standardise and harmonise assessment methods between European countries, and increase the accuracy with which intake of SF is measured, it is essential that (1) an agreed method and approach to portion size estimation is used and (2) this is used in conjunction with a standardised source of composition data. This review has indicated five instruments, all FFQs (Food4me, PURE, IMMIDIET, HAPIEE, and FFQ used by Piqueras et al.) which meet both criteria, and were tested for validity to assess SF intake. These instruments may be most suitable to assess intake of SF among healthy populations across Europe. These methods have been used in pan-European populations which encompass a range of European regions, and should be considered by future studies which focus on evaluating SF intake. However, these instruments have only been tested for validity in one country. Future work is needed to test the validity of these instruments across European countries.

## Additional file

Additional file 1: EMBASE search strategy. (PNG 62 kb)

## Abbreviations

24-HDR: 24 Hour Dietary Recall
CVD: Cardiovascular disease
DD: 7-day diet diary
DD: Diet Diary
DEDIPAC: DEterminants of DIet and Physical Activity
DR: Dietary Record
EFSA: European Food Safety Authority
EPIC: European Prospective Investigation into Cancer and Nutrition
EYHS: European Youth Heart Study
F&V: Fruit and vegetable
FCD: Food composition database
FCT: Food composition table
FFQ: Food Frequency Questionnaire
HAPIEE: Health, Alcohol and Psychosocial factors in Eastern Europe
HDL: High-density lipoprotein
HELENA: Healthy Lifestyle in Europe by Nutrition in Adolescence
ICC: Intraclass correlation coefficient
IDAMES: Innovative Dietary Assessment Methods in Epidemiological Studies and Public Health
IDEFICS: Dietary- and lifestyle-induced health EFfects In Children and infantS
LDL: Low-density lipoprotein
MUFA: Monounsaturated Fatty Acids
PRISMA: Preferred Reporting Items for Systematic Reviews and Meta-Analyses
PUFA: Polyunsaturated Fatty Acids
PURE: Prospective Urban Rural Epidemiology
SACINA: Self-Administered Children and Infants Nutrition Assessment
SENECA: Survey in Europe on Nutrition and the Elderly; a Concerted Action
SF: Saturated Fat
SFA: Saturated Fatty Acids
SSBs: Sugar-sweetened beverages
WFR: Weighed Food Record
WHO: World Health Organisation
WHO-MONICA: The World Health Organization Multinational MONItoring of trends and determinants in CArdiovascular disease
ZENITH: Zinc Effects on Nutrient/nutrient Interactions and Trends in Health and Aging
