# Supplementary figures and images for: A systematic review of methods to assess intake of saturated fat (SF) among healthy European adults and children: a DEDIPAC (Determinants of Diet and Physical Activity) study
Source: BMC Nutr. 2018 May 8;4:21. doi: 10.1186/s40795-018-0231-1 (PMC7050932; doi:10.1186/s40795-018-0231-1)

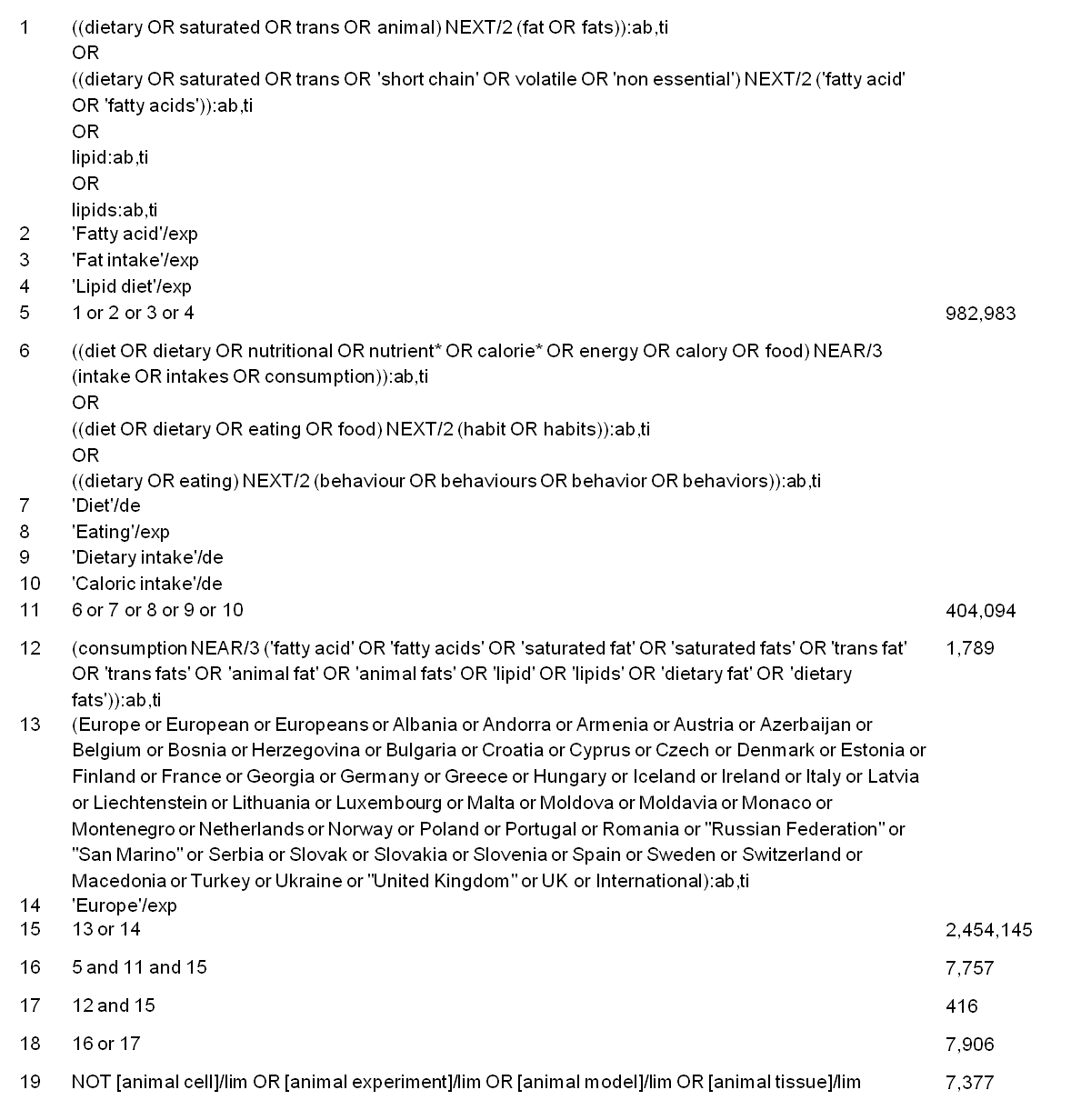

Supplement: Supplementary file 1 — EMBASE search strategy. (PNG 62 kb) [file 40795_2018_231_MOESM1_ESM.png]
